# Bis(μ-cyclo­hexane-1,4-dicarboxyl­ato)bis­{aqua­[1-(1*H*-imidazo[4,5-*f*][1,10]phenanthrolin-2-yl)naphthalen-2-ol]cadmium} monohydrate

**DOI:** 10.1107/S1600536811004727

**Published:** 2011-02-12

**Authors:** Xiu-Yan Wang, Shuai Ma, Yu He

**Affiliations:** a College of Chemistry, Jilin Normal University, Siping 136000, People’s Republic of China, and Key Laboratory of Preparation and Applications of Environmentally Friendly Materials (Jilin Normal University), Ministry of Education, People’s Republic of China

## Abstract

The asymmetric unit of the title compound, [Cd_2_(C_8_H_10_O_4_)_2_(C_23_H_14_N_4_O)_2_(H_2_O)_2_]·H_2_O, consists of one half of the dimeric complex, which lies about an inversion centre, and a half-occupancy solvent water mol­ecule on a general position. Each Cd^II^ cation is six-coordinated by the two N atoms from one 1-(1*H*-imidazo[4,5-*f*][1,10]phenanthrolin-2-yl)naphthalen-2-ol (*L*) ligand and three O atoms from two different 1,4-chdc^2−^ ligands (1,4-H_2_chdc = cyclo­hexane-1,4-dicarb­oxy­lic acid), two coordinating in a bidentate fashion and the other in a monodentate fashion. The distorted octa­hedral coordination sphere is completed by a coordinated water mol­ecule. The Cd^II^ atoms are each bridged by two 1,4-chdc^2−^ ligands, forming an inversion dimer with the *L* ligands located on the outside of the dimeric unit. An intra­molecular N—H⋯O hydrogen bond occurs. In the crystal, O—H⋯O and N—H⋯O hydrogen-bonding inter­actions stabilize the packing.

## Related literature

For background to the coordination chemistry of 1,10-phenanthroline and its derivatives, see: Wang *et al.* (2010 ▶).
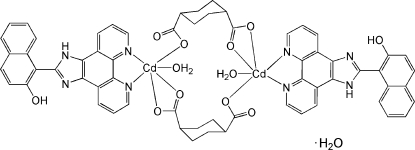


## Experimental

### 

#### Crystal data

[Cd_2_(C_8_H_10_O_4_)_2_(C_23_H_14_N_4_O)_2_(H_2_O)_2_]·H_2_O
*M* *_r_* = 1342.92
Triclinic, 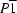

*a* = 9.870 (3) Å
*b* = 11.871 (4) Å
*c* = 12.459 (4) Å
α = 66.788 (4)°
β = 86.066 (4)°
γ = 87.462 (4)°
*V* = 1338.2 (7) Å^3^
*Z* = 1
Mo *K*α radiation
μ = 0.87 mm^−1^
*T* = 293 K
0.21 × 0.18 × 0.16 mm

#### Data collection

Bruker APEX diffractometer
Absorption correction: multi-scan (*SADABS*; Sheldrick, 1996 ▶) *T*_min_ = 0.41, *T*_max_ = 0.64
6918 measured reflections
4657 independent reflections
4195 reflections with *I* > 2σ(*I*)
*R*_int_ = 0.012

#### Refinement

*R*[*F* ^2^ > 2σ(*F* ^2^)] = 0.026
*wR*(*F* ^2^) = 0.066
*S* = 1.04
4657 reflections
400 parameters
H atoms treated by a mixture of independent and constrained refinement
Δρ_max_ = 0.40 e Å^−3^
Δρ_min_ = −0.36 e Å^−3^

### 

Data collection: *SMART* (Bruker, 1997 ▶); cell refinement: *SAINT* (Bruker, 1999 ▶); data reduction: *SAINT*; program(s) used to solve structure: *SHELXS97* (Sheldrick, 2008 ▶); program(s) used to refine structure: *SHELXL97* (Sheldrick, 2008 ▶); molecular graphics: *SHELXTL* (Sheldrick, 2008 ▶); software used to prepare material for publication: *SHELXL97*.

## Supplementary Material

Crystal structure: contains datablocks global, I. DOI: 10.1107/S1600536811004727/sj5103sup1.cif

Structure factors: contains datablocks I. DOI: 10.1107/S1600536811004727/sj5103Isup2.hkl

Additional supplementary materials:  crystallographic information; 3D view; checkCIF report

## Figures and Tables

**Table 1:** Hydrogen-bond geometry (Å, °)

*D*—H⋯*A*	*D*—H	H⋯*A*	*D*⋯*A*	*D*—H⋯*A*
N4—H4⋯O5	0.86	1.93	2.513 (3)	124
O5—H5⋯O3^i^	0.75 (3)	1.81 (3)	2.546 (3)	167 (3)
O1*W*—H*W*12⋯O2^ii^	0.79 (4)	1.96 (4)	2.738 (4)	171 (4)
O1*W*—H*W*11⋯O2	0.79 (5)	2.49 (5)	3.059 (4)	130 (4)
O1*W*—H*W*11⋯O5^iii^	0.79 (5)	2.51 (5)	3.118 (3)	135 (4)

Symmetry codes: (i) 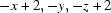; (ii) 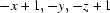; (iii) 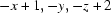.

**Fig. 1.**
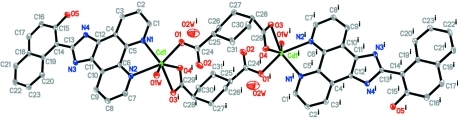
The structure of (I), showing the atomic numbering scheme with displacement ellipsoids drawn at the 30% probability level. Hydrogen atoms have been omitted for clarity. [Symmetry codes: (i) 2-x, -y, 1-z]

## supplementary crystallographic information

### Comment

The coordination chemistry of 1,10-phenanthroline-like ligands has generated considerable recent interest (Wang *et al.*, 2010). 1-(1H-imidazo[4,5-f][1,10]phenanthrolin-2-yl)naphthalen-2-ol (L), is a good candidate as a N-donor ligand, as it has excellent coordinating ability. In this work, we selected 1,4-H_2_chdc^2-^ ligand (1,4-H_2_chdc = cyclohexane-1,4-dicarboxylic acid) as an organic linker and L as an N-donor chelating ligand, to generate a new Cd^II^ complex, [Cd_2_(L)_2_(1,4-chdc)_2_(H_2_O)_2_]^.^H_2_O.

The asymmetric unit of the title compound, (I), consists of one half of the dimeric complex, which lies about an inversion centre, and a half occupancy solvent water molecule which occupies a general position.  Each  Cd^II^ cation is six-coordinated by the N1 & N2 atoms from one L ligand (L = 1-(1H-imidazo[4,5-f][1,10]phenanthrolin-2-yl)naphthalen-2-ol), the O1, O3 and O4 atoms from two different 1,4-chdc^2-^ ligands (1,4-H_2_chdc = cyclohexane-1,4-dicarboxylic acid), O3 and O4 coordinating in a bidentate fashion with O1  monodentate. The distorted octahedral coordination sphere is completed by the O1W atom of a coordinated water molecule.  In the crystal structure O-H···O and N-H···O H-bonding interactions, Table 1, stabilize the packing.

### Experimental

A mixture of CdCl_2_^.^2.5H_2_O (0.5 mmol), 1,4-H_2_chdc (0.5 mmol) and L (0.5 mmol) in 10 mL distilled water was heated at 460 K in a Teflon-lined stainless steel autoclave for seven days. The reaction system was then slowly cooled to room temperature. Pale yellow crystals of (I) suitable for single crystal X-ray diffraction analysis were collected from the final reaction system by filtration, washed several times with distilled water and dried in air at ambient temperature. Yield: 29% based on Cd(II).

### Refinement

All H atoms on C and N atoms were positioned geometrically (N-H = 0.86 Å and C-H = 0.93 Å) and refined as riding, with U_iso_(H)=1.2U_eq_(carrier). The water H-atoms of O1W were located in difference Fourier maps, and were refined freely. However, the hydrogen atoms of the half occupancy water molecule were not located in difference Fourier maps.

### Crystal data

**Table:**

[Cd_2_(C_8_H_10_O_4_)_2_(C_23_H_14_N_4_O)_2_(H_2_O)_2_]·H_2_O	*Z* = 1
*M**_r_* = 1342.92	*F*(000) = 681
Triclinic, *P*1	*D*_x_ = 1.666 Mg m^−^^3^
Hall symbol: -P 1	Mo *K*α radiation, λ = 0.71073 Å
*a* = 9.870 (3) Å	Cell parameters from 4657 reflections
*b* = 11.871 (4) Å	θ = 1.9–25.2°
*c* = 12.459 (4) Å	µ = 0.87 mm^−^^1^
α = 66.788 (4)°	*T* = 293 K
β = 86.066 (4)°	Block, pale yellow
γ = 87.462 (4)°	0.21 × 0.18 × 0.16 mm
*V* = 1338.2 (7) Å^3^	

### Data collection

**Table:**

Bruker APEX diffractometer	4657 independent reflections
Radiation source: fine-focus sealed tube	4195 reflections with *I* > 2σ(*I*)
graphite	*R*_int_ = 0.012
φ and ω scans	θ_max_ = 25.2°, θ_min_ = 1.9°
Absorption correction: multi-scan (*SADABS*; Sheldrick, 1996)	*h* = −11→11
*T*_min_ = 0.41, *T*_max_ = 0.64	*k* = −13→14
6918 measured reflections	*l* = −7→14

### Refinement

**Table:**

Refinement on *F*^2^	Primary atom site location: structure-invariant direct methods
Least-squares matrix: full	Secondary atom site location: difference Fourier map
*R*[*F*^2^ > 2σ(*F*^2^)] = 0.026	Hydrogen site location: inferred from neighbouring sites
*wR*(*F*^2^) = 0.066	H atoms treated by a mixture of independent and constrained refinement
*S* = 1.04	*w* = 1/[σ^2^(*F*_o_^2^) + (0.0338*P*)^2^ + 0.5714*P*] where *P* = (*F*_o_^2^ + 2*F*_c_^2^)/3
4657 reflections	(Δ/σ)_max_ = 0.001
400 parameters	Δρ_max_ = 0.40 e Å^−^^3^
0 restraints	Δρ_min_ = −0.36 e Å^−^^3^

### Special details

**Table:**

Geometry. All esds (except the esd in the dihedral angle between two l.s. planes) are estimated using the full covariance matrix. The cell esds are taken into account individually in the estimation of esds in distances, angles and torsion angles; correlations between esds in cell parameters are only used when they are defined by crystal symmetry. An approximate (isotropic) treatment of cell esds is used for estimating esds involving l.s. planes.
Refinement. Refinement of F^2^ against ALL reflections. The weighted R-factor wR and goodness of fit S are based on F^2^, conventional R-factors R are based on F, with F set to zero for negative F^2^. The threshold expression of F^2^ > 2sigma(F^2^) is used only for calculating R-factors(gt) etc. and is not relevant to the choice of reflections for refinement. R-factors based on F^2^ are statistically about twice as large as those based on F, and R- factors based on ALL data will be even larger.

### Fractional atomic coordinates and isotropic or equivalent isotropic displacement parameters (Å^2^)

**Table:**

	*x*	*y*	*z*	*U*_iso_*/*U*_eq_	Occ. (<1)
Cd1	0.616015 (18)	0.060066 (16)	0.681838 (16)	0.03702 (8)	
C14	0.3661 (2)	0.4379 (2)	1.1701 (2)	0.0327 (5)	
N1	0.6509 (2)	0.05928 (18)	0.86655 (18)	0.0359 (5)	
C11	0.4036 (2)	0.3089 (2)	0.9525 (2)	0.0320 (5)	
C19	0.2604 (2)	0.5310 (2)	1.1481 (2)	0.0351 (6)	
N3	0.3363 (2)	0.38774 (18)	0.99551 (19)	0.0351 (5)	
C12	0.5066 (2)	0.2464 (2)	1.0227 (2)	0.0325 (5)	
O3	1.36041 (17)	−0.21386 (17)	0.50727 (16)	0.0434 (4)	
O1W	0.4350 (2)	−0.0379 (2)	0.6417 (2)	0.0485 (5)	
O5	0.5359 (2)	0.3191 (2)	1.2950 (2)	0.0529 (5)	
N4	0.5031 (2)	0.28855 (18)	1.11010 (18)	0.0354 (5)	
H4	0.5566	0.2661	1.1668	0.043*	
O4	1.18245 (19)	−0.18121 (18)	0.40333 (16)	0.0482 (5)	
C29	1.2364 (2)	−0.2339 (2)	0.4977 (2)	0.0341 (5)	
O1	0.7412 (2)	−0.10599 (18)	0.72164 (19)	0.0572 (5)	
C4	0.5946 (2)	0.1583 (2)	1.0002 (2)	0.0335 (5)	
N2	0.4511 (2)	0.1855 (2)	0.72357 (19)	0.0388 (5)	
C28	1.1619 (3)	−0.3238 (2)	0.6062 (2)	0.0417 (6)	
H28	1.2225	−0.3952	0.6395	0.050*	
C13	0.3986 (2)	0.3733 (2)	1.0911 (2)	0.0330 (5)	
C5	0.5733 (2)	0.1395 (2)	0.8980 (2)	0.0321 (5)	
C6	0.4653 (2)	0.2070 (2)	0.8212 (2)	0.0330 (5)	
C25	0.8894 (2)	−0.2291 (2)	0.6558 (2)	0.0378 (6)	
H25	0.8691	−0.2996	0.7290	0.045*	
C10	0.3794 (2)	0.2901 (2)	0.8494 (2)	0.0334 (5)	
C15	0.4384 (2)	0.4081 (2)	1.2701 (2)	0.0375 (6)	
C3	0.6987 (3)	0.0894 (2)	1.0710 (2)	0.0414 (6)	
H3	0.7152	0.0989	1.1395	0.050*	
C18	0.2375 (3)	0.5914 (2)	1.2272 (2)	0.0382 (6)	
C30	1.0302 (3)	−0.3707 (3)	0.5831 (3)	0.0528 (8)	
H30A	1.0046	−0.4443	0.6502	0.063*	
H30B	1.0463	−0.3930	0.5162	0.063*	
C16	0.4145 (3)	0.4692 (3)	1.3466 (3)	0.0440 (6)	
H16	0.4655	0.4481	1.4120	0.053*	
O2	0.7087 (3)	−0.1025 (3)	0.5475 (2)	0.0881 (9)	
C17	0.3173 (3)	0.5587 (2)	1.3248 (3)	0.0457 (7)	
H17	0.3031	0.5991	1.3752	0.055*	
C24	0.7707 (3)	−0.1388 (2)	0.6379 (3)	0.0436 (6)	
C1	0.7484 (3)	−0.0044 (2)	0.9354 (2)	0.0431 (6)	
H1	0.8009	−0.0599	0.9139	0.052*	
C26	1.0203 (3)	−0.1744 (3)	0.6697 (3)	0.0443 (6)	
H26A	1.0060	−0.1441	0.7315	0.053*	
H26B	1.0448	−0.1056	0.5976	0.053*	
C7	0.3521 (3)	0.2430 (3)	0.6540 (3)	0.0479 (7)	
H7	0.3423	0.2277	0.5873	0.058*	
C21	0.1335 (3)	0.6827 (2)	1.2073 (3)	0.0475 (7)	
H21	0.1201	0.7227	1.2582	0.057*	
C8	0.2622 (3)	0.3256 (3)	0.6773 (3)	0.0519 (7)	
H8	0.1937	0.3641	0.6269	0.062*	
C31	0.9114 (3)	−0.2774 (3)	0.5593 (3)	0.0486 (7)	
H31A	0.9299	−0.2094	0.4851	0.058*	
H31B	0.8291	−0.3160	0.5535	0.058*	
C20	0.1736 (3)	0.5655 (3)	1.0538 (3)	0.0465 (7)	
H20	0.1855	0.5279	1.0008	0.056*	
C2	0.7751 (3)	0.0085 (2)	1.0378 (2)	0.0450 (6)	
H2	0.8444	−0.0375	1.0835	0.054*	
C9	0.2756 (3)	0.3495 (2)	0.7742 (2)	0.0421 (6)	
H9	0.2164	0.4046	0.7906	0.051*	
C27	1.1360 (3)	−0.2690 (3)	0.6991 (2)	0.0517 (7)	
H27A	1.1145	−0.3344	0.7744	0.062*	
H27B	1.2182	−0.2307	0.7055	0.062*	
C22	0.0534 (3)	0.7124 (3)	1.1158 (3)	0.0546 (8)	
H22	−0.0144	0.7724	1.1042	0.066*	
C23	0.0727 (3)	0.6528 (3)	1.0385 (3)	0.0560 (8)	
H23	0.0167	0.6726	0.9764	0.067*	
O2W	0.9490 (10)	−0.0713 (7)	0.2643 (8)	0.152 (3)	0.50
H5	0.561 (3)	0.295 (3)	1.356 (3)	0.048 (10)*	
HW12	0.386 (4)	−0.001 (3)	0.592 (3)	0.060 (12)*	
HW11	0.472 (5)	−0.090 (4)	0.625 (4)	0.111 (18)*	

### Atomic displacement parameters (Å^2^)

**Table:**

	*U*^11^	*U*^22^	*U*^33^	*U*^12^	*U*^13^	*U*^23^
Cd1	0.03780 (12)	0.03642 (12)	0.03781 (12)	0.00468 (8)	0.00499 (8)	−0.01731 (9)
C14	0.0305 (12)	0.0311 (13)	0.0377 (14)	−0.0019 (10)	0.0047 (10)	−0.0156 (11)
N1	0.0367 (11)	0.0300 (11)	0.0392 (12)	0.0052 (9)	0.0063 (9)	−0.0137 (9)
C11	0.0327 (12)	0.0284 (12)	0.0371 (13)	0.0014 (10)	0.0042 (10)	−0.0163 (11)
C19	0.0345 (13)	0.0305 (13)	0.0402 (14)	−0.0041 (10)	0.0086 (11)	−0.0151 (11)
N3	0.0340 (11)	0.0325 (11)	0.0422 (12)	0.0049 (9)	0.0002 (9)	−0.0191 (10)
C12	0.0344 (12)	0.0315 (13)	0.0332 (13)	−0.0001 (10)	0.0040 (10)	−0.0152 (11)
O3	0.0332 (9)	0.0511 (11)	0.0413 (10)	−0.0014 (8)	0.0006 (8)	−0.0136 (9)
O1W	0.0520 (13)	0.0472 (12)	0.0519 (13)	0.0006 (10)	−0.0018 (11)	−0.0257 (11)
O5	0.0503 (12)	0.0695 (14)	0.0514 (13)	0.0228 (10)	−0.0181 (11)	−0.0371 (12)
N4	0.0334 (11)	0.0390 (12)	0.0374 (12)	0.0074 (9)	−0.0029 (9)	−0.0195 (10)
O4	0.0448 (11)	0.0589 (12)	0.0374 (11)	−0.0027 (9)	−0.0047 (9)	−0.0146 (10)
C29	0.0367 (13)	0.0322 (13)	0.0378 (14)	0.0042 (10)	0.0031 (11)	−0.0198 (11)
O1	0.0623 (13)	0.0473 (12)	0.0633 (14)	0.0190 (10)	0.0024 (11)	−0.0264 (11)
C4	0.0302 (12)	0.0316 (13)	0.0366 (14)	0.0013 (10)	0.0047 (10)	−0.0125 (11)
N2	0.0399 (12)	0.0424 (12)	0.0389 (12)	0.0057 (10)	0.0004 (10)	−0.0223 (10)
C28	0.0345 (13)	0.0335 (14)	0.0488 (16)	0.0088 (11)	0.0044 (12)	−0.0093 (12)
C13	0.0315 (12)	0.0315 (13)	0.0370 (14)	−0.0014 (10)	0.0051 (10)	−0.0155 (11)
C5	0.0322 (12)	0.0266 (12)	0.0351 (13)	−0.0009 (10)	0.0078 (10)	−0.0110 (10)
C6	0.0337 (12)	0.0284 (12)	0.0353 (13)	−0.0006 (10)	0.0059 (10)	−0.0121 (11)
C25	0.0328 (13)	0.0340 (13)	0.0436 (15)	0.0014 (10)	0.0032 (11)	−0.0131 (12)
C10	0.0340 (13)	0.0299 (12)	0.0356 (13)	0.0001 (10)	0.0040 (10)	−0.0131 (11)
C15	0.0322 (13)	0.0405 (14)	0.0441 (15)	−0.0007 (11)	0.0032 (11)	−0.0221 (12)
C3	0.0430 (14)	0.0421 (15)	0.0370 (14)	0.0051 (12)	0.0000 (12)	−0.0145 (12)
C18	0.0402 (14)	0.0311 (13)	0.0444 (15)	−0.0056 (11)	0.0111 (12)	−0.0178 (12)
C30	0.0441 (15)	0.0369 (15)	0.081 (2)	−0.0043 (12)	0.0143 (15)	−0.0295 (15)
C16	0.0442 (15)	0.0504 (16)	0.0449 (16)	−0.0055 (13)	−0.0003 (12)	−0.0267 (14)
O2	0.0831 (18)	0.111 (2)	0.0816 (18)	0.0567 (16)	−0.0378 (15)	−0.0501 (17)
C17	0.0539 (16)	0.0430 (15)	0.0509 (17)	−0.0057 (13)	0.0092 (14)	−0.0312 (14)
C24	0.0358 (14)	0.0378 (14)	0.0567 (18)	0.0027 (11)	0.0033 (13)	−0.0193 (14)
C1	0.0428 (15)	0.0357 (14)	0.0491 (16)	0.0106 (12)	0.0044 (13)	−0.0170 (13)
C26	0.0382 (14)	0.0574 (17)	0.0456 (16)	0.0024 (12)	0.0019 (12)	−0.0301 (14)
C7	0.0503 (16)	0.0559 (18)	0.0447 (16)	0.0137 (14)	−0.0087 (13)	−0.0279 (14)
C21	0.0514 (16)	0.0386 (15)	0.0561 (18)	0.0003 (12)	0.0147 (14)	−0.0253 (14)
C8	0.0519 (17)	0.0604 (19)	0.0505 (18)	0.0216 (14)	−0.0163 (14)	−0.0296 (15)
C31	0.0335 (13)	0.0531 (17)	0.072 (2)	0.0000 (12)	−0.0013 (13)	−0.0381 (16)
C20	0.0512 (16)	0.0433 (16)	0.0483 (16)	0.0128 (13)	−0.0021 (13)	−0.0231 (13)
C2	0.0436 (15)	0.0411 (15)	0.0462 (16)	0.0141 (12)	−0.0050 (12)	−0.0138 (13)
C9	0.0399 (14)	0.0422 (15)	0.0475 (16)	0.0117 (12)	−0.0043 (12)	−0.0222 (13)
C27	0.0359 (14)	0.080 (2)	0.0354 (15)	0.0092 (14)	−0.0004 (12)	−0.0205 (15)
C22	0.0536 (18)	0.0425 (16)	0.066 (2)	0.0158 (14)	0.0079 (16)	−0.0229 (15)
C23	0.0563 (18)	0.0518 (18)	0.0589 (19)	0.0204 (14)	−0.0070 (15)	−0.0224 (15)
O2W	0.196 (8)	0.118 (6)	0.146 (7)	0.012 (6)	−0.077 (7)	−0.048 (5)

### Geometric parameters (Å, °)

**Table:**

Cd1—O1	2.1806 (19)	C6—C10	1.405 (3)
Cd1—N2	2.328 (2)	C25—C24	1.519 (4)
Cd1—N1	2.346 (2)	C25—C31	1.522 (4)
Cd1—O3^i^	2.3474 (19)	C25—C26	1.522 (4)
Cd1—O1W	2.358 (2)	C25—H25	0.9800
Cd1—O4^i^	2.431 (2)	C10—C9	1.404 (4)
Cd1—C29^i^	2.750 (3)	C15—C16	1.410 (4)
C14—C15	1.392 (4)	C3—C2	1.367 (4)
C14—C19	1.444 (3)	C3—H3	0.9300
C14—C13	1.480 (3)	C18—C17	1.408 (4)
N1—C1	1.331 (3)	C18—C21	1.419 (4)
N1—C5	1.355 (3)	C30—C31	1.538 (4)
C11—C12	1.375 (3)	C30—H30A	0.9700
C11—N3	1.376 (3)	C30—H30B	0.9700
C11—C10	1.425 (3)	C16—C17	1.358 (4)
C19—C20	1.418 (4)	C16—H16	0.9300
C19—C18	1.431 (3)	O2—C24	1.232 (4)
N3—C13	1.325 (3)	C17—H17	0.9300
C12—N4	1.364 (3)	C1—C2	1.388 (4)
C12—C4	1.430 (3)	C1—H1	0.9300
O3—C29	1.280 (3)	C26—C27	1.523 (4)
O3—Cd1^i^	2.3474 (19)	C26—H26A	0.9700
O1W—HW12	0.79 (4)	C26—H26B	0.9700
O1W—HW11	0.79 (5)	C7—C8	1.394 (4)
O5—C15	1.353 (3)	C7—H7	0.9300
O5—H5	0.75 (3)	C21—C22	1.354 (4)
N4—C13	1.374 (3)	C21—H21	0.9300
N4—H4	0.8600	C8—C9	1.361 (4)
O4—C29	1.238 (3)	C8—H8	0.9300
O4—Cd1^i^	2.431 (2)	C31—H31A	0.9700
C29—C28	1.518 (4)	C31—H31B	0.9700
C29—Cd1^i^	2.750 (3)	C20—C23	1.372 (4)
O1—C24	1.263 (3)	C20—H20	0.9300
C4—C5	1.407 (4)	C2—H2	0.9300
C4—C3	1.408 (4)	C9—H9	0.9300
N2—C7	1.328 (3)	C27—H27A	0.9700
N2—C6	1.355 (3)	C27—H27B	0.9700
C28—C30	1.524 (4)	C22—C23	1.401 (4)
C28—C27	1.538 (4)	C22—H22	0.9300
C28—H28	0.9800	C23—H23	0.9300
C5—C6	1.468 (3)		
			
O1—Cd1—N2	154.70 (8)	C31—C25—C26	109.6 (2)
O1—Cd1—N1	90.33 (8)	C24—C25—H25	107.3
N2—Cd1—N1	71.31 (7)	C31—C25—H25	107.3
O1—Cd1—O3^i^	118.02 (7)	C26—C25—H25	107.3
N2—Cd1—O3^i^	87.26 (7)	C9—C10—C6	118.0 (2)
N1—Cd1—O3^i^	132.00 (7)	C9—C10—C11	124.2 (2)
O1—Cd1—O1W	90.15 (9)	C6—C10—C11	117.8 (2)
N2—Cd1—O1W	86.39 (8)	O5—C15—C14	119.8 (2)
N1—Cd1—O1W	124.30 (7)	O5—C15—C16	118.3 (2)
O3^i^—Cd1—O1W	95.35 (8)	C14—C15—C16	121.9 (2)
O1—Cd1—O4^i^	89.09 (8)	C2—C3—C4	119.2 (3)
N2—Cd1—O4^i^	108.12 (8)	C2—C3—H3	120.4
N1—Cd1—O4^i^	91.54 (7)	C4—C3—H3	120.4
O3^i^—Cd1—O4^i^	54.35 (6)	C17—C18—C21	120.6 (2)
O1W—Cd1—O4^i^	144.16 (7)	C17—C18—C19	119.7 (2)
O1—Cd1—C29^i^	103.41 (8)	C21—C18—C19	119.7 (3)
N2—Cd1—C29^i^	99.92 (8)	C28—C30—C31	113.8 (2)
N1—Cd1—C29^i^	113.53 (7)	C28—C30—H30A	108.8
O3^i^—Cd1—C29^i^	27.66 (7)	C31—C30—H30A	108.8
O1W—Cd1—C29^i^	120.39 (8)	C28—C30—H30B	108.8
O4^i^—Cd1—C29^i^	26.75 (7)	C31—C30—H30B	108.8
C15—C14—C19	118.2 (2)	H30A—C30—H30B	107.7
C15—C14—C13	119.5 (2)	C17—C16—C15	120.1 (3)
C19—C14—C13	122.3 (2)	C17—C16—H16	120.0
C1—N1—C5	118.8 (2)	C15—C16—H16	120.0
C1—N1—Cd1	124.85 (16)	C16—C17—C18	121.2 (2)
C5—N1—Cd1	116.05 (16)	C16—C17—H17	119.4
C12—C11—N3	110.9 (2)	C18—C17—H17	119.4
C12—C11—C10	120.9 (2)	O2—C24—O1	123.6 (3)
N3—C11—C10	128.2 (2)	O2—C24—C25	121.1 (3)
C20—C19—C18	116.8 (2)	O1—C24—C25	115.3 (3)
C20—C19—C14	124.2 (2)	N1—C1—C2	122.9 (2)
C18—C19—C14	118.9 (2)	N1—C1—H1	118.6
C13—N3—C11	105.0 (2)	C2—C1—H1	118.6
N4—C12—C11	105.4 (2)	C25—C26—C27	111.3 (2)
N4—C12—C4	130.9 (2)	C25—C26—H26A	109.4
C11—C12—C4	123.7 (2)	C27—C26—H26A	109.4
C29—O3—Cd1^i^	93.97 (15)	C25—C26—H26B	109.4
Cd1—O1W—HW12	121 (3)	C27—C26—H26B	109.4
Cd1—O1W—HW11	103 (3)	H26A—C26—H26B	108.0
HW12—O1W—HW11	108 (4)	N2—C7—C8	122.4 (3)
C15—O5—H5	116 (2)	N2—C7—H7	118.8
C12—N4—C13	107.5 (2)	C8—C7—H7	118.8
C12—N4—H4	126.2	C22—C21—C18	121.2 (3)
C13—N4—H4	126.2	C22—C21—H21	119.4
C29—O4—Cd1^i^	91.14 (15)	C18—C21—H21	119.4
O4—C29—O3	120.3 (2)	C9—C8—C7	119.5 (3)
O4—C29—C28	123.0 (2)	C9—C8—H8	120.3
O3—C29—C28	116.7 (2)	C7—C8—H8	120.3
O4—C29—Cd1^i^	62.11 (14)	C25—C31—C30	111.5 (2)
O3—C29—Cd1^i^	58.37 (13)	C25—C31—H31A	109.3
C28—C29—Cd1^i^	173.23 (18)	C30—C31—H31A	109.3
C24—O1—Cd1	116.31 (19)	C25—C31—H31B	109.3
C5—C4—C3	118.0 (2)	C30—C31—H31B	109.3
C5—C4—C12	116.2 (2)	H31A—C31—H31B	108.0
C3—C4—C12	125.9 (2)	C23—C20—C19	121.8 (3)
C7—N2—C6	119.1 (2)	C23—C20—H20	119.1
C7—N2—Cd1	124.19 (17)	C19—C20—H20	119.1
C6—N2—Cd1	116.56 (16)	C3—C2—C1	119.4 (3)
C29—C28—C30	114.4 (2)	C3—C2—H2	120.3
C29—C28—C27	110.7 (2)	C1—C2—H2	120.3
C30—C28—C27	110.5 (2)	C8—C9—C10	119.4 (2)
C29—C28—H28	106.9	C8—C9—H9	120.3
C30—C28—H28	106.9	C10—C9—H9	120.3
C27—C28—H28	106.9	C26—C27—C28	112.2 (2)
N3—C13—N4	111.2 (2)	C26—C27—H27A	109.2
N3—C13—C14	126.9 (2)	C28—C27—H27A	109.2
N4—C13—C14	121.9 (2)	C26—C27—H27B	109.2
N1—C5—C4	121.8 (2)	C28—C27—H27B	109.2
N1—C5—C6	117.5 (2)	H27A—C27—H27B	107.9
C4—C5—C6	120.8 (2)	C21—C22—C23	119.9 (3)
N2—C6—C10	121.6 (2)	C21—C22—H22	120.0
N2—C6—C5	117.8 (2)	C23—C22—H22	120.0
C10—C6—C5	120.6 (2)	C20—C23—C22	120.6 (3)
C24—C25—C31	113.5 (2)	C20—C23—H23	119.7
C24—C25—C26	111.5 (2)	C22—C23—H23	119.7

Symmetry codes: (i) −*x*+2, −*y*, −*z*+1.

### Hydrogen-bond geometry (Å, °)

**Table:**

*D*—H···*A*	*D*—H	H···*A*	*D*···*A*	*D*—H···*A*
N4—H4···O5	0.86	1.93	2.513 (3)	124
O5—H5···O3^ii^	0.75 (3)	1.81 (3)	2.546 (3)	167 (3)
O1W—HW12···O2^iii^	0.79 (4)	1.96 (4)	2.738 (4)	171 (4)
O1W—HW11···O2	0.79 (5)	2.49 (5)	3.059 (4)	130 (4)
O1W—HW11···O5^iv^	0.79 (5)	2.51 (5)	3.118 (3)	135 (4)

Symmetry codes: (ii) −*x*+2, −*y*, −*z*+2; (iii) −*x*+1, −*y*, −*z*+1; (iv) −*x*+1, −*y*, −*z*+2.
